# Cytotoxic activity of Thai medicinal plants against human cholangiocarcinoma, laryngeal and hepatocarcinoma cells *in vitro*

**DOI:** 10.1186/1472-6882-10-55

**Published:** 2010-09-28

**Authors:** Wiratchanee Mahavorasirikul, Vithoon Viyanant, Wanna Chaijaroenkul, Arunporn Itharat, Kesara Na-Bangchang

**Affiliations:** 1 Graduate Program in Biomedical Sciences, Thammasat University (Rangsit Campus), Pathumtani 12121, Thailand; 2 Applied Thai Traditional Medicine Center, Thammasart University (Rangsit Campus), Pathumtani 12121, Thailand

## Abstract

**Background:**

Cholangiocarcinoma is a serious public health in Thailand with increasing incidence and mortality rates. The present study aimed to investigate cytotoxic activities of crude ethanol extracts of a total of 28 plants and 5 recipes used in Thai folklore medicine against human cholangiocarcinoma (CL-6), human laryngeal (Hep-2), and human hepatocarcinoma (HepG2) cell lines in vitro.

**Methods:**

Cytotoxic activity of the plant extracts against the cancerous cell lines compared with normal cell line (renal epithelial cell: HRE) were assessed using MTT assay. 5-fluorouracil was used as a positive control. The IC_50 _(concentration that inhibits cell growth by 50%) and the selectivity index (SI) were calculated.

**Results:**

The extracts from seven plant species (*Atractylodes lancea*, *Kaempferia galangal*, *Zingiber officinal*, *Piper chaba*, *Mesua ferrea*, *Ligusticum sinense*, *Mimusops elengi*) and one folklore recipe (Pra-Sa-Prao-Yhai) exhibited promising activity against the cholangiocarcinoma CL-6 cell line with survival of less than 50% at the concentration of 50 μg/ml. Among these, the extracts from the five plants and one recipe (*Atractylodes lancea*, *Kaempferia galangal*, *Zingiber officinal*, *Piper chaba*, *Mesua ferrea*, and Pra-Sa-Prao-Yhai recipe) showed potent cytotoxic activity with mean IC_50 _values of 24.09, 37.36, 34.26, 40.74, 48.23 and 44.12 μg/ml, respectively. All possessed high activity against Hep-2 cell with mean IC_50 _ranging from 18.93 to 32.40 μg/ml. In contrast, activity against the hepatoma cell HepG2 varied markedly; mean IC_50 _ranged from 9.67 to 115.47 μg/ml. The only promising extract was from *Zingiber officinal *(IC_50 _= 9.67 μg/ml). The sensitivity of all the four cells to 5-FU also varied according to cell types, particularly with CL-6 cell (IC_50 _= 757 micromolar). The extract from *Atractylodes lancea *appears to be both the most potent and most selective against cholangiocarcinoma (IC_50 _= 24.09 μg/ml, SI = 8.6).

**Conclusions:**

The ethanolic extracts from five plants and one folklore recipe showed potent cytotoxic activity against CL-6 cell. Sensitivity to other cancerous cell lines varied according to cell types and the hepatocarcinoma cell line. HepG2 appears to be the most resistant to the tested extracts.

## Background

Cholangiocarcinoma, malignant epithelial cells that arises within bile duct, is a serious public health in Thailand with increasing incidence and mortality. The cancer occurs at a particularly high rate in Northeastern Thailand, with age-standardized incidence rate (ASRs) 33.4 *per *100,000 in males and 12.3 *per *100,000 in females [1]. It accounts for approximately 15% of liver cancer worldwide [2]. This cancer can be classified into three major groups, *i.e*., intrahepatic, perihilar and distal extrahepatic cholangiocarcinoma. Intrahepatic type is the most common case of cholangiocarcinoma in Thailand and infestation of *Opisthorchis viverrini *has been classified as a definite risk factor of the disease [3]. The lack of early detection and limited therapeutic options are major problems for controlling this type of cancer. At present, surgical resection of detectable tumors leads to an improvement in the 5-year survival rate. Adjunctive therapy with chemotherapeutic agents has been shown to improve local control, provide palliation, and prolong survival [4]. Even those with operable tumor, the recurrence rate is extremely high, with a 5-year survival rate of less than 40% [5,6]. Chemotherapeutic treatment of cholangiocarcinoma is largely ineffective; the standard chemotherapeutic agent, 5-fluorouracil (5-FU) always produces low clinical response rate [6-8]. Advanced surgical techniques in conjunction with alternative chemotherapeutic option with promising activity are required to improve the survival of patients. Cholangiocarcinoma is considered to be a multidrug and radio-resistant tumor and still require new approach of treatments [9].

Numerous cancer research for chemotherapeutic potential of medicinal plants have been carried out in an effort to discover new therapeutic agents that lack the toxic effects associated with current therapeutic agents. Traditional medicine is commonly used as an alternative treatment for cancer by Thai people [10]. Several Thai traditional folklores have been shown to possess anticancer activities in various human cancerous cell lines with some promising candidates [11,12]. In the present study, the ethanolic extracts of a total of 28 plants and 5 recipes used in Thai folklore medicine were investigated for their cytotoxic activity *in vitro *against three human cancerous cell lines, *i.e*., CL-6 (cholangiocarcinoma), Hep-2 (laryngeal carcinoma), HepG2 (hepatocarcinoma), and HRE (renal epithelial cells). To our knowledge, this is the first study that focused on the investigation of cytotoxic activity of Thai folklore against cholangiocarcinoma.

## Methods

### Reagents

Commercial grade ethanol was purchased from Labscan Co. Ltd. The cell culture medium and reagents were purchased from different sources: Ham-12, RPMI 1640, M-199 medium, from Gibco BRL Life Technologies (Grand Island, NY, USA), renal epithelium cell growth medium and SupplementPack from Promacell Co. Ltd. (Germany). Fetal bovine serum (FBS), L-glutamine dimethylsulfoxide (DMSO), the antibiotic solution, streptomycin-penicillin and antibiotic-antimycotic, were purchased from Gibco BRL Life Technologies. The reference compound, 5-fluorouracil (5-FU) and MTT [3-(4,5-dimethylthiazol-2-yl)-2,5-diphenyltetrazolium bromide] were purchased from Sigma-Aldrich Inc. (St. Louis, MO, USA).

### Plant materials and preparation of crude extracts

Plant materials were collected from various parts of Thailand and some were purchased from the city markets. Authentication of plant materials was carried out at the herbarium of the Department of Forestry, Bangkok, Thailand, where the herbarium vouchers have been kept. A duplicate set has been deposited in the herbarium of Southern Center of Thai Medicinal Plants at the Faculty of Pharmaceutical Science, Prince of Songkhla University, Songkhla, Thailand.

The plant materials were rinsed thoroughly with tap water to remove extraneous contaminants and cut into small pieces, oven-dried at 50°C until stability of dry weight was observed, and then ground into powder with an electric-grinder. Extraction was carried out by macerating the powdered plant materials (100 g) in stoppered flasks containing 500 ml of 95% ethanol at room temperature (25-30°C) for 7 days. The extracted solvent was separated and filtered through Whatman no. 1 filter paper. After filtration, the extracts were evaporated under reduced pressure by rotary evaporation. The crude extracts were weighed and stored at −20°C until used.

### Preparation of test materials and reference drug

The crude extract from each medicinal plant/recipe was initially dissolved in 50% ethanol. Concentrated stock solution of each extract was prepared by adding a known weight of each crude extract to a known volume of 50% ethanol, and then serially diluted (1:2) with complete media to obtain the working solutions at eight final concentrations. Positive control agent 5-FU was prepared similarly as the crude extract by dissolving in 50% ethanol.

### *In vitro *assay for cytotoxic activity

#### Human cell lines and cell culture

The cholangiocarcinoma cell line CL-6, human laryngeal carcinoma cell line Hep-2, human hepatocarcinoma cell line HepG2 and normal human epithelial cell (HRE) were used for cytotoxic screening of the medicinal plant extracts. CL-6 cell line was established and kindly provided by Associate Professor Dr. Adisak Wongkajornsilp, Department of Pharmacology, Faculty of Medicine (Siriraj Hospital), Mahidol University, and were cultured in Ham-12 medium supplemented with 10% heated fetal bovine serum and 100 IU/ml of antibiotic-antimycotic solution. Hep-2 cell line was obtained from Department of Medical Technology, Faculty of Allied Health Sciences, Thammasat University, established and cultured in M-199 medium supplemented with 10% heated fetal bovine serum, 2.5 mM HEPES (pH 7.4) and 100 IU/ml penicillin-streptomycin solution. HepG2 cell line was purchased from the Cell Line Service Co. Ltd. (Germany) and was cultured in DMEM: Ham's F12 medium supplemented with 2 mM L-glutamine, 10% fetal bovine serum and 100 IU/ml pen-strep. Normal human renal epithelial cell line (HRE) was purchased from Promocell Co. Ltd. (Germany) and cultured in renal epithelial cell growth medium 2 with SupplementPack. All cells were maintained at 37°C in a 5% CO_2 _atmosphere with 95% humidity.

#### Cytotoxic assay

The MTT colorimetric assay developed by Mosmann [13] with modification was used to screen for cytotoxic activity of all the plant extracts. Briefly, the cells were seeded in 96-well plates at a density of 10^4 ^cells/well in 100 μl culture medium. Following 24-h incubation and attachment, the cells were treated with different concentrations of plant extracts and 5-FU (positive control) for 24 h. Each extract was screened initially for its cytotoxicity against all cancerous and normal cell lines at the concentration of 50 μg/ml. The potential candidates which resulted in cell survival of less than 50% were further assessed for their IC_50 _(concentration that inhibits cell growth by 50%) values at the concentration range of 250 and 1.95 μg/ml. The concentration range used for 5-FU was 78.13 to 10,000 μM. Following washing and incubation with MTT solution (20 μl of 5 mg/ml) at 37°C for 3 h, cells were lyzed with DMSO. The yellow MTT dye was reduced by succinic dehydrogenase in the mitochondria of viable cells to purple formazan crystals. Absorbance (OD) was measured at 570 nm using a microplate reader (Varioscan Flash, Thermo, Finland). The percentage of cytotoxicity compared to the untreated cells was determined with the equation:

$$\text{Cell viability }\left(\%\right)\;\;=\frac{\text{OD of treated cells}}{\text{OD of control cells}}\times \text{1}00$$

The results were generated from three independent experiments; each experiment was performed in triplicate. The IC_50 _values were calculated using CalcySyn™ (USA) software. The selectivity index (SI) was also calculated from the IC_50 _ratio of normal epithelial and cancerous (CL-6, Hep-2, HepG2) cells. SI value indicates selectivity of the sample to the cell lines tested. Samples with SI value greater than 3 were considered to have high selectivity.

## Results and Discussion

Plants are promising source of anti-infective and anticancer chemotherapeutic agents. Saetung and colleague selected twelve Thai medicinal plants as the ingredients of a Southern Thai traditional folklore medicine for cancer treatment to test for their cytotoxicity activity against human lung and prostate cancer cell lines. The ethanolic extracts of the six plants (*Bridelia ovata, Curcuma zedoaria*, *Derris scandens*, *Dioscorea membranacea*, *Nardostachys jatamansi *and *Rhinacanthus nasutus*) showed promising cytotoxic activity (IC_50 _< 30 μg/ml) [14]. In the present study, the cytotoxic effect of a total of 28 ethanolic extracts of plants and 5 recipes from traditional folklore medicine against the human cholangiocarcinoma cell line CL-6 were investigated. In addition, their comparative activity against the other two human cancerous cell lines, *i.e*., Hep-2 (laryngeal carcinoma) and HepG2 (hepatocarcinoma), and one normal cell HRE (human renal epithelial cell) was also examined. These plants have been used by Thai people to treat different illness. The five recipes were used for restorativeness, treatment of fever, cold and cancer (Table 1). The eighteen plants tested were composition of Pra-Sa-Prao-Yhai recipe, and some plants were composition of other recipes (Table 1). The leave, stem, bark and rhizome parts of plants were most often used for these traditional medicines (Table 1). Results from the initial screening showed that the extracts from seven plant species (*Atractylodes lancea*, *Kaempferia galangal, Zingiber officinal, Piper chaba, Mesua ferrea*, *Ligusticum sinense, Mimusops elengi*) and one folklore recipe (Pra-Sa-Prao-Yhai) exhibited promising activity against the cholangiocarcinoma CL-6 cell line with cell survival of less than 50% at the concentration of 50 μg/ml (Table 2). Among these, six (*Atractylodes lancea*, *Kaempferia galangal, Zingiber officinal, Piper chaba, Mesua ferrea*, and Pra-Sa-Prao-Yhai recipe) showed potent cytotoxic activity with IC_50 _values of less than 50 μg/ml. The potency of the extracts in descending order was as follow: *Atractylodes lancea*, *Zingiber officinal*, *Kaempferia galangal*, *Piper chaba*, Pra-Sa-Prao-Yhai recipe and *Mesua ferrea *(Table 3). The plants *Atractylodes lancea*, *Kaempferia galangal *and *Mesua ferrea *are also composition of Pra-Sa-Prao-Yhai recipe. In the US National Cancer Institute Plant Screening Program, a crude extract is generally considered to have *in vitro *cytotoxic activity if the IC_50 _value in carcinoma cells, following incubation between 48 and 72 hours, is less than 20 μg/ml, while it is less than 4 μg/ml for pure compounds [15]. Based on this criteria, only the extract from *Atractylodes lancea *is considered highly active with IC_50 _of 24.04 ± 3.40 (mean ± SD) μg/ml. The extract from *Dioscorea membranacea *showed only weak activity in the screening test against cholangiocarcinoma cell. In our previous study [11], water extract of this plant (DM1 and DM2) exhibited promising cytotoxic activity against human breast adenocarcinoma MCF-7 (IC_50 _= 7.7 μg/ml) but only showed moderate activity against human large cell lung carcinoma COR-L23 (IC_50 _= 37.6 μg/ml) and human colon adenocarcinoma LS-174T (IC_50 _= 78.4 μg/ml) cell lines [16].

**Table 1:** The plant species and recipes used in Thai traditional folklore which were investigated for cytotoxicity

Family	Plant	Part used	Voucher specimen	Thai traditional Use
Compositae	*Artemisia annua L*.^1^	Rh	SKP 051010101	Treatment of fever, hemorrhoids [31]
Compositae	*Atractylodes lancea (thung.) DC*.^1^	Rh	SKP 051011201	Treatment of fever, colds, flu, sore throat [31]
Cruciferae	*Asclepias curassavica L*.^1,4^	Fl	SKP 057121901	Used as analgesic [32]
Dioscoreaceae	*Dioscorea membranacea*	Rh	SKP 062041305	Treatment of cancer [16]
Dracaenaceae	*Dracaena loureiri Gagnep*.^1^	St, Ba	SKP 065041201	Treatment of cough, fever, inflammation [32]
Guttiferae	*Mammea siamensis Kosterm*^1^	Fl	SKP 083131901	Restorative [31]
Guttiferae	*Mesua ferrea L*^1^	Fl	SKP 083130601	Treatment of dyspepsia [31]
Myristicaceae	*Myristica fragrans Houtt*.^1^	Sd	SKP 121130601	Treatment of uterus pain, diarrhea [33]
Myrtaceae	*Syzygium aromaticum (L.) Merr. & L.M. Perry*^1^	Fl	SKP 123190101	Treatment of toothache, bacterial infection [32]
Nelumbonaceae	*Nigella sativa Linn*.^1,4^	Sd	SKP 160141901	Treatment of jaundice [32]
Piperaceae	*Piper chaba Linn*^2,3^	Fr	SKP 146160301	Used as carminative, antidiarrheal [31]
Piperaceae	*Piper interruptum Opiz*. ^2,3^	Lf	SKP 146160901	Treatment of choke [31]
Piperaceae	*Piper sarmentosum *Roxb.^2,3^	Rt	SKP 146161901	Treatment of fever, toothache, cough, asthma [31]
Plumbaginaceae	*Plumbago indica Linn*.^2,3^	Rt	SKP 148160901	Treatment of rheumatism [32]
Smilacaceae	*Smilax corbularia Kunth*	Rh	SKP 179190315	Treatment of cancer [16]
Sapotadeae	*Mimusops elengi L*.^1^	Fl	SKP 171130501	Used as cordial, tonic. Treatment of syncope [32]
Umbelliferae	*Angelica dahurica Benth*.^1^	Rt	SKP 199010401	Used as antipyretic, antiasthma, anticough [33]
Umbelliferae	*Angelica sinensis (Oliv.) Diels*^1^	Rh	SKP 199010901	Treatment of bronchitis pleurisy [33]
Umbelliferae	*Anethum graveolens L*.^1,4^	Rt, Fr	SKP 199010701	Used as carminative. Treatment of eye pain [32]
Umbelliferae	*Cuminum cyminum Linn*.^1,4^	Sd	SKP 199030301	Treatment of dyspepsia, diarrhoea and jaundice [31]
Umbelliferae	*Foeniculum vulgare Mill. var. dulce Alef*.^1,4^	Sd	SKP 199062201	Used as analeptic [33]
Umbelliferae	*Ligusticum sinense Oliv. cv. Chuanxiong*^1^	Rh	SKP 199121901	Treatment of urinary bladder channel, headache, neurodermatitis [32]
Zingiberaceae	*Amomum testaceum Ridl*.^1^	Sd	SKP 206011101	Used as carminative, antibacterial [33]
Zingiberaceae	*Curcuma longa Linn*.	Rh	SKP 206012101	Treatment of cancer, high cholesterol, dyspepsia, gallstone [33]
Zingiberaceae	*Kaempferia galangal*^1^	Lf	SKP 206110701	Antinociceptive, anti-inflammatory [33]
Zingiberaceae	*Zingiber officinale *Roscoe.^2^	Rh	SKP 206261501	Treatment of hypercholesteremia and high level triglyceride [33]
Zingiberaceae	*Zingiber ligulatum Roxb^.3^*	Rh	SKP 206261201	Used as anti-inflammatory [33]
-	*Dioscorea membranacea *&*Smilax corbularia*	-	-	Treatment of cancer [16]
-	Ben-ja-Kul 1 Recipe	-	-	Used as restorative [31]
-	Ben-ja-Kul 2 Recipe	-	-	Treatment of fever, cold [31]
-	Pra-Sa-Prao-Yhai Recipe	-	-	Used as restorative, anti-emetic, setting up proper digestive system, analeptic [31]
-	Tein-5 Recipe	-	-	Used as restorative, carminative [31]

Plant parts: Fr: Fruits, Fl: Flower, Lf: leaves, Rt: Root, Rh: Rhizomes, Sd: Seed, St: Stem.^1^Composition in Pra-Sa-Prao-Yhai Recipe, ^2^Composition in Ben-ja-Kul 1 Recipe, ^3^Composition in Ben-ja-Kul 2 Recipe, ^4^Composition in Tein-5 Recipe

**Table 2:** Percentage survival of cancer cell lines (CL-6, HepG2, Hep-2) treated with ethanolic extract from a total of 28 plants and 5 recipes used in Thai folklore medicine at the concentration of 50 μg/ml

Plant	Cell line		
	CL-6	HepG2	Hep-2
*Atractylodes lancea*	32.10 ± 3.72	93.80 ± 8.09	-1.38 ± 0.67
*Mesua ferrea*	35.67 ± 8.66	64.62 ± 2.92	12.55 ± 6.03
*Kaempferia galangal (leaf)*	36.74 ± 11.72	75.03 ± 17.78	5.65 ± 0.08
*Ligusticum sinense Oliv*	43.85 ± 6.73	69.74 ± 4.46	36.81 ± 15.56
*Zingiber officinal*	44.26 ± 8.93	80.65 ± 11.92	8.11 ± 10.97
*Artemisia annua*	46.56 ± 6.03	95.76 ± 11.35	33.48 ± 7.85
*Kaempferia galangal (flower)*	47.49 ± 5.95	69.36 ± 16.12	9.74 ± 0.07
*Mimusops elengi*	48.84 ± 0.62	109.99 ± 2.95	54.44 ± 3.24
Pra-Sa-Prao-Yhai Recipe	49.43 ± 8.75	79.55 ± 23.90	25.55 ± 5.32
*Piper chaba*	50.62 ± 3.10	72.25 ± 1.15	12.42 ± 7.99
*Mammea siamensis*	51.35 ± 3.89	29.17 ± 12.28	59.52 ± 44.80
*Angelica sinensis*	51.77 ± 7.53	81.94 ± 12.48	33.77 ± 9.19
*Syzygium aromaticum*	55.40 ± 0.51	72.51 ± 15.32	34.30 ± 21.33
*Nigella sativa*	58.02 ± 2.25	118.49 ± 6.33	43.68 ± 0.49
*Curcuma longa*	59.86 ± 6.49	13.87 ± 12.88	0.57 ± 0.80
Ben-ja-Kul 1 Recipe	61.33 ± 2.84	87.19 ± 6.98	33.12 ± 9.68
*Foeniculum vulgare*	61.62 ± 8.86	83.26 ± 4.78	50.55 ± 11.04
*Anethum graveolens*	63.28 ± 12.92	97.47 ± 7.40	55.11 ± 4.37
Ben-ja-Kul 2 Recipe	63.78 ± 5.15	93.02 ± 9.15	46.27 ± 4.00
*Piper pendulispicum*	64.94 ± 5.78	62.66 ± 16.28	25.60 ± 14.67
*Myristica fragrans*	66.11 ± 2.11	89.14 ± 20.97	33.00 ± 13.32
*Piper sarmentosum*	69.20 ± 4.39	81.95 ± 10.79	34.09 ± 8.96
*Amomum testaceum*	72.30 ± 11.24	90.74 ± 12.47	71.24 ± 2.10
*Cuminum cyminum*	74.07 ± 6.38	87.25 ± 19.06	39.28 ± 7.50
*Zingiber ligulatum*	74.68 ± 2.17	106.55 ± 14.23	44.14 ± 2.28
*Dioscorea membranacea*	76.05 ± 2.57	90.86 ± 16.65	59.90 ± 29.93
*Plumbago indica*	77.79 ± 14.31	61.83 ± 20.45	40.50 ± 13.52
Tein-5 Recipe	79.73 ± 2.56	89.61 ± 16.60	63.21 ± 3.74
*Dracaena loureiri*	81.29 ± 10.42	96.18 ± 5.81	20.97 ± 28.05
*Asclepias curassavica*	81.63 ± 3.99	87.47 ± 13.67	47.45 ± 5.01
*Smilax corbularia*	81.77 ± 9.61	147.02 ± 18.39	68.96 ± 13.38
*Dioscorea membranacea *&*Smilax corbularia*	82.31 ± 0.39	141.96 ± 14.64	76.64 ± 13.42
*Angelica dahurica*	85.16 ± 5.55	80.18 ± 13.41	48.68 ± 2.68

Data are presented as mean ± SD from 3 independent experiments, triplicate for each)

**Table 3:** Cytotoxicity of 5-FU and ethanolic extracts from 5 medicinal plants and one recipe with promising activity

Plants	Cell line						
	CL-6		HepG2		Hep-2		HRE
	IC_50_	SI	IC_50_	SI	IC_50_	SI	IC_50_
*Atractylodes lancea*	24.09 ± 3.40	8.6	76.68 ± 15.94	2.7	29.35 ± 8.66	7.1	207.59 ± 7.97
*Kaempferia galangal *(leaf)	37.36 ± 3.98	2.9	115.47 ± 26.23	0.9	18.99 ± 10.33	5.7	119.20 ± 14.91
*Zingiber officinal*	34.26 ± 7.65	3.5	9.67 ± 3.91	12.6	32.40 ± 6.70	3.8	121.50 ± 5.20
*Piper chaba*	40.74 ± 5.30	4.5	68.09 ± 22.58	2.7	18.93 ± 5.03	9.8	119.14 ± 9.94
Pra-Sa-Prao-Yhai recipe	44.12 ± 11.58	5.9	125.07 ± 3.08	2.11	20.99 ± 2.68	12.5	263.51 ± 29.06
*Mesua ferrea*	48.23 ± 5.84	2.5	86.47 ± 4.38	1.4	19.22 ± 5.31	6.3	121.77 ± 29.08
5-FU (μM)	757.00 ± 77.16	2.0	633.08 ± 284.25	2.4	141.49 ± 17.14	10.8	1542.20 ± 1529.11

Data are presented otherwise specified as mean ± SD of IC_50 _(μg/ml) from 3 independent experiments, triplicate for each.

**Figure 1:**
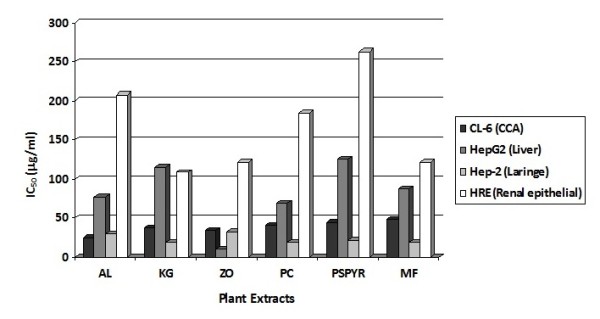
**Mean IC_50 _values of the ethanolic extracts from 5 plants and one recipe against 3 cancerous cell lines (CL-6, HepG2, Hep-2) and one normal cell (HRE)**: AL = *Atractylodes lancea*, KG *= Kaempferia galangal*, ZO *= Zingiber officinal*, PC *= Piper chaba*, PSPYR *= Pra-Sa-Prao-Yhai recipe*, MF *= Mesua ferrea*

Investigation of comparative cytotoxic activities of the extracts of the six plants and the standard drug 5-FU against CL-6, HepG-2 Hep-2 and HRE cell lines indicate difference in responsiveness/sensitivity of different cancerous cells to these plant extracts (Table 3 and Figure 1). The results were generally in agreement with that shown in the screening test, confirming that Hep-2 was the most sensitive, while HepG2 was the most resistant cell line to the tested ethanolic extracts from Thai traditional folklore. All extracts possessed high activity against Hep-2 cell with mean IC_50 _values ranging from 18.93 to 32.40 μg/ml. In contrast, activity against the hepatoma cell HepG2 varied markedly with mean IC_50 _values ranging from 9.67 to 115.47 μg/ml. The only promising extract was from *Zingiber officinal *(IC_50 _= 9.67 ± 3.91 μg/ml). The extract from *Atractylodes lancea *exhibited the most potent activity against CL-6 but the activities against the other two cancerous cells were only moderate. The extract from *Zingiber officinal *was most promising against HepG2 cell line, whereas that from *Piper chaba *was most promising against Hep-2 cell. This may suggest that HepG2 is the most resistant among the three cancerous cell lines under investigation. Only two crude extracts from *Curcuma longa *and *Mammea siamensis *showed high activity against HepG2 cell. Crude extracts from *Curcuma longa *exhibited high activity against both HepG2 and Hep-2 (% survival of 13.9 and 0.6, respectively) but relatively low activity against CL-6 cell line (% survival of 59.9). The ethanolic extract of *Mammea siamensis *showed promising activity against only HepG2 cell (29.2%) with relatively low activity against CL-6 and Hep-2 cell line with % survival of 51.4 and 59.5, respectively (Table 2). Selectivity of the cytotoxic activity of the six tested extracts was determined by comparing the cytotoxic activity (IC_50_) of each plant extract against each cancerous cell with that of the normal human cell HRE (Table 3). Results were expressed as selectivity index (SI). SI of greater than 3 was considered as highly selective. The extract from *Atractylodes lancea *appears to be both the most potent and most selective against cholangiocarcinoma (IC_50 _= 24.09 ± 3.40 μg/ml, SI 8.6), whereas that from *Zingiber officinal *appears to be the most potent and most selective against HepG2 (IC_50 _= 9.67 ± 3.91 μg/ml, SI = 12.6). For Hep-2 cell, *Piper chaba *(IC_50 _= 18.63 ± 5.03 μg/ml, SI = 9.8) and Pra-Sa-Prao-Yhai recipe (IC_50 _= 20.99 ± 2.68 μg/ml, SI = 12.5) exhibited the most promising and most selective cytotoxic activity.

Difference in responsiveness/sensitivity of different cancerous cells to different chemotherapeutics are commonly observed in various studies [11,12,17,18]. The responsiveness of all the four cells to 5-FU varied according to cell types, particularly with CL-6 cell (IC_50 _= 757 ± 72.16 μM). Although 5-FU is the standard chemotherapeutic drug used in the treatment of human cholangiocarcinoma, evidence of 5-FU resistance has been reported both *in vitro *[9] and *in vivo *[7]. It has been demonstrated in a previous study [19] that variations in the sensitivity to chemotherapeutic drugs were observed among the five intrahepatic cholangiocarcinoma cell lines and sensitivity to chemotherapeutic drug. Cholangiocarcinoma is a fatal disease which is highly resistant to anticancer drugs. It is noted that the IC_50 _values of 5-FU observed in the present study and the previous study [19] were much higher than those reported in other cancerous cell lines, *e.g*., colon carcinoma (HCC-48, COLO20) [20] and cervical squamous carcinoma (SiHa, HeLa) cell lines [21]. This suggests low sensitivity of cholangiocarcinoma to all chemotherapeutics including those obtained from medicinal plant source, which is probably due in part to enhanced resistance to apoptosis. Several genes involved in the apoptosis, detoxification and efflux processes have been reported to influence resistance to chemotherapeutic agents, for examples multidrug resistance protein (MDR1) and multidrug-resistance associated proteins (MRPs) [22], glutathione-S-transferase (GST) [23], dihydropyrimidine dehydrogenase (DPD) [24], and galectin-3 [25]. Active compound from *Atractylodes lancea *has been shown to exhibit strong inhibitory effects on 5-lipoxygenase (5-LOX) and cyclooxygenase-1 (COX-1), but exhibited only weak antioxidative activities [IC_50 _= 0.1 μM (5-LOX), 2 μM (COX-1), 9 μM (PMN/FMLP), 28 μM (PMN/OZ)] [26]. The alcoholic extracts of *Kaempferia galangal *showed high cytotoxicity against SW 620 with IC_50 _less than 30 μg/ml and showed moderate cytotoxicity against cancer cells DU145 (human prostate cancer cell line), PA1 (human ovarian teratocarcinoma cell line), and B16F10 (murine melanoma cells) and were not selective against cancer cells when compared to Vero cells [27]. In the case of *Zingiber officinal *methanolic extracts, cytotoxic activities against human A549 (adenocarcinomic alveolar basal epithelial cell line), SK-OV-3 (ovarian carcinoma cell line), SK-MEL-2 (skin Melanoma cell line), and HCT15 (colon carcinoma cell line) have been reported [28]. From the study of Sakpakdeejaroen *et al*., piperine, the active compound of *Piper chaba*, showed cytotoxic activity against MCF-7 (breast cancer cell line) with IC_50 _equal to 35.72 μM [29]. The active compounds of *Mesua ferrea *including phenylcoumarins, xanthones and triterpenoids were reported to possess cytotoxic and antibacterial activities [30].

## Conclusions

Results obtained from this study indicate that 6 out of a total of 28 plants and 5 recipes (*Atractylodes lancea*, *Kaempferia galangal, Zingiber officinal, Piper chaba, Mesua ferrea*, and Pra-Sa-Prao-Yhai recipe) used in Thai folklore medicine exhibited promising cytotoxic activity against CL-6 human cholangiocarcinoma cell line. Sensitivity to other cancerous cell lines varied according to cell types and the hepatocarcinoma HepG2 appears to be the most resistant cell line to the tested extracts. The extract from *Atractylodes lancea *appears to be both the most potent and most selective against cholangiocarcinoma, whereas that from *Zingiber officinal *appears to be the most potent and most selective against HepG2. The extract from *Piper chaba *(IC_50 _= 18.63 μg/ml, SI = 9.8) and Pra-Sa-Prao-Yhai recipe (IC_50 _= 20.99 μg/ml, SI = 12.5.) exhibited the most promising and most selective cytotoxic activity against Hep-2 cell line. Further investigation of all the six extracts for their cytotoxic activity against cholangiocarcinoma in hamster model is underway to fully assess the anticancer activity *in vivo*.

## Competing interests

The authors declare that they have no competing interests.

## Authors' contributions

KN conceived and designed the study, reviewed and finalized the manuscript. WM performed the laboratory work, and drafted the manuscript. VV obtained the financial support for the project, reviewed and provided comments and suggestions to improve the quality of the manuscript. WC performed data analysis. AI prepared the medicinal plants and all the herbal extracts. All authors read and approved the final manuscript.

## Pre-publication history

The pre-publication history for this paper can be accessed here:

http://www.biomedcentral.com/1472-6882/10/55/prepub
